# Influence of oat components on lipid digestion using an *in vitro* model: Impact of viscosity and depletion flocculation mechanism

**DOI:** 10.1016/j.foodhyd.2018.05.018

**Published:** 2018-10

**Authors:** Myriam M.L. Grundy, David J. McClements, Simon Ballance, Peter J. Wilde

**Affiliations:** a Quadram Institute Bioscience, Norwich Research Park, Colney, Norwich NR4 7UA, UK; b University of Reading, School of Agriculture, Policy and Development, Earley Gate, Reading RG6 6AR, UK; c Biopolymers and Colloids Laboratory, Department of Food Science, University of Massachusetts, Amherst, MA 01003, USA; d Nofima, Norwegian Institute for Food, Fisheries and Aquaculture Research, PB 210, N-1431 Ås, Norway

**Keywords:** Oat β-glucan, Flocculation, Viscosity, Molecular weight, Lipolysis, BG1, oat β-glucan of high Mw, BG2, oat β-glucan of medium Mw, BG3, oat β-glucan of low Mw, CFC, critical flocculation concentration, CVC, critical viscosity concentration, FFA, free fatty acids, Mw, weight-average molecular weight, *R*_*h*_, weight-average hydrodynamic radius, WPI, whey protein isolate

## Abstract

Depletion flocculation is a well-known instability mechanism that can occur in oil-in-water emulsions when the concentration of non-adsorbed polysaccharide exceeds a certain level. This critical flocculation concentration depends on the molecular characteristics of the polysaccharide molecules, such as their molecular weight and hydrodynamic radius. In this study, a range of analytical methods (dynamic shear rheology, optical microscopy, and static light-scattering) were used to investigate the interaction between lipid droplets and polysaccharides (guar gum and β-glucans) of varying weight-average molecular weight and hydrodynamic radius, and concentration. The aim of this work was to see if the health benefits of soluble fibers like β-glucans could be explained by their influence on the structure and digestibility of lipid emulsions. The apparent viscosity of the emulsions increased with increasing polysaccharide concentration, molecular weight, and hydrodynamic radius. Droplet flocculation was observed in the emulsions only at certain polysaccharide concentrations, which was attributed to a depletion effect. In addition, the water-soluble components in oat flakes, flour, and bran were extracted using aqueous solutions, to examine their impact on emulsion stability and properties. Then, the rate and extent of lipolysis of a sunflower oil-in-water emulsion in the presence of these oat extracts were monitored using the pH-stat method. However, the inhibition of lipolysis was not linearly related to the viscosity of the oat solutions. The water-soluble extracts of β-glucan collected from oat flakes had a significant inhibitory effect on lipolysis. The results of this study increase our understanding of the possible mechanisms influencing the impact of oat constituents on lipid digestion. This work also highlights the importance of considering the molecular properties of polysaccharides, and not just their impact on solution viscosity.

## Highlights

• Smaller β-glucans induce depletion flocculation over wider concentration range.
• β-glucan molecules occupied the bulk solution around the droplets and drove them together.
• Viscosity is not the only factor affecting depletion flocculation.
• Compounds released from oat flakes caused the greatest depletion flocculation.
• Lipolysis was reduced in the presence of oat flake extract solutions.

## Graphical abstract

The effect of oat and β-glucan on lipid digestion relies on complex mechanisms.

**Figure:**
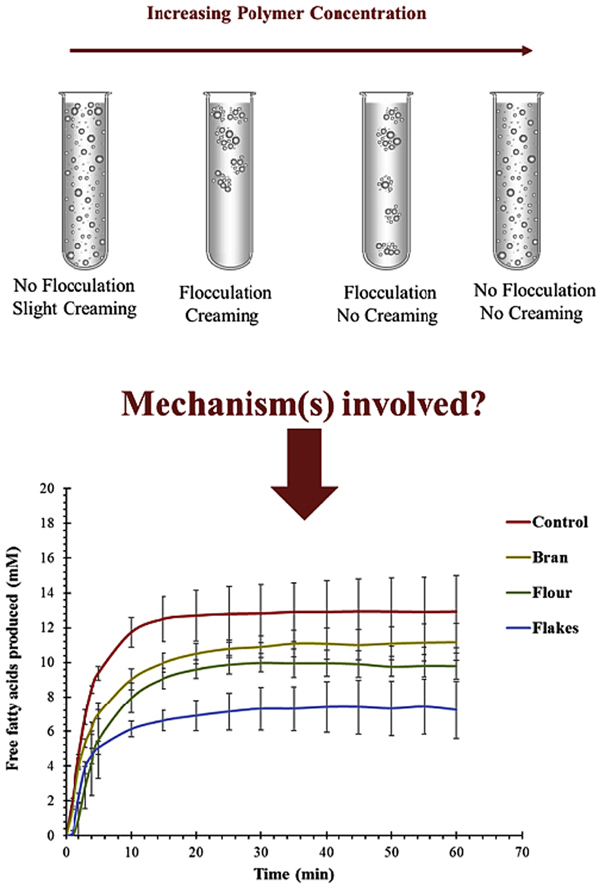

## 1 Introduction

The ability of oat (*Avena sativa* L.) to affect lipid metabolism and blood cholesterol levels is now well-known even though the mechanisms involved are not fully understood (Grundy, Fardet, Tosh, Rich, & Wilde, 2018). Oats contain a range of constituents that may positively impact human health, especially water-soluble polysaccharides such as β-glucan (Martínez-Villaluenga & Peñas, 2017; Miller & Fulcher, 2011). This type of polysaccharide may inhibit lipid digestion due to its ability to increase viscosity or promote droplet flocculation, which reduces the access of lipase to the oil droplet surfaces (Bai et al., 2017; Grundy, Quint, Rieder, Ballance, Dreiss, Cross, et al., 2017). Consequently, the presence of these soluble dietary fibres in foods could benefit human health by modulating the blood lipid levels after ingestion of foods rich in lipids. However, there is currently a poor understanding of the precise molecular and physicochemical mechanisms by which dietary fibres inhibit lipid digestion.

In the late 1990s, it was shown experimentally that neutral non-adsorbing polymers could promote droplet flocculation in oil-in-water emulsions through a depletion mechanism (Jenkins & Snowden, 1996). The tendency for depletion flocculation to occur depends on the molecular weight (Mw) and hydrodynamic radius (*R*_*h*_) of the polymer molecules, which has been described mathematically using theoretical models (Asakura & Oosawa, 1954, 1958). Non-adsorbed polymers induce flocculation in emulsions through an osmotic effect. In an emulsion containing non-adsorbing polymers, there is a region surrounding each droplet where the polymer concentration is depleted (depletion zone). As a result, there is an osmotic pressure between the depletion zone and the bulk polymer solution. It is energetically favourable to minimise the osmotic potential differences in the system, so the system will tend towards a state where the total volume of the depletion zones is minimised. Therefore, when two lipid droplets approach each other so that their depletion zones overlap, there is a reduction in the total volume of solution from which the polymers are excluded, which is energetically favourable. Thus, the system tends towards droplet association and drives flocculation. The magnitude of the osmotic pressure increases with increasing polymer concentration, and so depletion flocculation can happen when the attractive forces outweigh the repulsive forces in the system (McClements, 2000).

Droplet flocculation often promotes more rapid gravitational separation (creaming) in an emulsion because the particle size is effectively increased. However, creaming may not be observed in some cases, because the viscosity of the solution also increases with increasing polymer concentration. For a particular polymer preparation, there is a critical concentration (c*) above which polymer entanglement occurs and a viscoelastic network is formed that restricts oil droplet movement (Sharafbafi, Alexander, Tosh, & Corredig, 2015; Syrbe, Bauer, & Klostermeyer, 1998). A number of experimental studies showed that the presence of different types of food-grade biopolymers in oil-in-water emulsions can induce depletion flocculation (Chung, Degner, & McClements, 2013; Espinal-Ruiz, Parada-Alfonso, Restrepo-Sanchez, Narvaez-Cuenca, & McClements, 2014; Minekus et al., 2005). The tendency for droplet flocculation and creaming to take place depends on the molecular characteristics and concentration of the polymers used, and has to be established for different kinds of polymers.

The present study was designed to establish the impact of polymer size and concentration on the viscosity and flocculation of oil-in-water emulsions, using common food-grade neutral polysaccharides (i.e., guar gum and β-glucan) with different molecular characteristics. Guar gum is a well characterised source of galactomannan that we used as a control. On the other hand, the β-glucan was selected because it is one of the main water-soluble polysaccharides found in oat, and has been proposed to be the cause of many of its health benefits, such as prevention of cardiovascular diseases, diabetes, obesity, cancer, and hypertension (Khan et al., 2018; Martínez-Villaluenga & Peñas, 2017; Rebello, O'Neil, & Greenway, 2016; Surampudi, Enkhmaa, Anuurad, & Berglund, 2016). In addition, water-soluble extracts isolated from oat flakes, flour, and bran (BG32) were collected to determine their impact on the stability of emulsions. Finally, we used these soluble extracts as a source of β-glucan and monitored their potential impact on lipid digestion using our *in vitro* duodenal model, in order to obtain some insights into the potential roles of polymer viscosity and depletion flocculation on free fatty acid (FFA) release. This study should provide some valuable insights into the molecular and physicochemical origin of the health benefits of soluble fibres in the human diet and complement some previous studies (Grundy, Quint, Rieder, Ballance, Dreiss, Butterworth, et al., 2017; Grundy, Quint, Rieder, Ballance, Dreiss, Cross, et al., 2017). Our main objective was therefore to fully characterise the materials used in this previous work, while investigating further how they influenced the emulsion stability. We believe that the innovation of the work presented here relies on more detailed characterisation steps that are often missing in the literature.

## 2 Materials and methods

### 2.1 Materials and samples characterisation

Sunflower oil, sodium chloride (99.8%), calcium chloride (99%), bovine bile extract, and pancreatin (40 U/mg of solid based on lipase activity) were purchased from Sigma-Adrich (Poole, Dorset, UK). High Mw oat β-glucan (BG1) was a generous gift from Dr Susan Tosh at Agricultural and Agri-Food Canada. Swedish Oat Fiber (Swedish Oat Fiber AB, Bua, Sweden) provided medium Mw β-glucan (BG2, brand name BG90) and the oat bran (brand name BG32). Low Mw oat β-glucan (BG3) was obtained from Megazyme (Bray, Wicklow, Ireland; Product Code: *P*-BGOM). Guar gum flour (Meyprogat M150) was provided by Dr Graham Sworn (Danisco, Paris, France). Oat flakes and oat flour were obtained as previously described (Grundy, Quint, Rieder, Ballance, Dreiss, Cross, et al., 2017). Powdered whey protein isolate (WPI) was donated by Davisco Foods International (Le Sueur, MN, USA).

The methods used for the determination of the moisture content, lipid content, polysaccharide concentrations of the oats (flakes, flour and bran), BG1, BG2, BG3, and guar gum are detailed elsewhere (Grundy, Quint, Rieder, Ballance, Dreiss, Butterworth, et al., 2017). Weight-, number-average molar mass, polydispersity, and weight-average *R*_*h*_ of purified β-glucan and galactomannan were determined by size-exclusion chromatography with a series coupled Wyatt 8 angle multi-angle light scattering detector, followed by a Wyatt Viscostar II viscosity detector, and finally a Wyatt T-rex refractive index detector (SEC-MALS-VISC-RI). For the oats flakes, flour and bran, the β-glucan was directly extracted and purified (omitting protease and xylanase treatment) as described by Rieder, Ballance, and Knutsen (2015). Briefly, 2 mg of purified sample was weighted into a 2 mL Eppendorf tube with screw lid. Twenty μL of 80% aqueous ethanol was added, vortexed, and left for 1 h with occasional mixing. To this 1.5 mL of 0.1 M sodium nitrate containing 0.02% sodium azide was added and the sample placed into a boiling water bath for 5 min followed by shaking at a frequency of 25s^-1^ in a Retch 400 M oscillating mill. This procedure of boiling and shaking was repeated a further time. Samples were finally filtered through a 0.8 μm syringe filter. One hundred μL of each sample was injected via a 100 μL loop onto two size-exclusion chromatography columns coupled in series (Tosho Bioscience TSK-gel PXWL 5000 and 6000). An isocratic mobile phase of 0.1 M sodium nitrate containing 0.02% sodium azide at a flow rate of 0.5 mL/min was used to elute the samples and delivered by a Shimadzu HPLC pump. Data was processed in custom Wyatt Astra software. The second virial coefficient was set at zero and a refractive index increment of 0.146 was used. As positive control, a certified pullulan standard of known molar mass from Polymer Standards Service was used. Treatment of samples/extracts containing β-glucan with lichenase followed by SEC-MALS-VISC-RI eliminated the concentration signal from the refractive index detector used to measure Mw in conjunction with the MALLS detector. This confirmed all the analysed sample/extracts comprised β-glucan. The amount of β-glucan released during the incubation of the oat materials were measured using an enzymatic method based on a cereal mixed-linkage β-glucan kit from Megazyme (Megazyme, Product Code: K-MBGL).

### 2.2 Preparation of the experimental material

Solutions of guar gum or β-glucan were obtained by slowly sprinkling the polymer powder into a rapidly swirling vortex of 10 mM phosphate buffer, pH 7. The mixture was heated at 80 °C for 2 h before being left at room temperature overnight. This procedure ensured that the polymers were fully hydrated. Additionally to those pure polymer preparations, water-soluble extracts from selected oat materials (flakes, flour or bran with a total β-glucan content of 1.0%, w/v) were incubated in 10 mM phosphate buffer as previously described (Grundy, Quint, Rieder, Ballance, Dreiss, Butterworth, et al., 2017). After 1 or 72 h of incubation, the samples were centrifuged at 1800 g for 10 min and the aqueous phase collected. This aqueous phase is referred as oat extract in the rest of the manuscript. Oat extracts were used in the present work in order to identify if the compounds/structures released during incubation, and not the oat particles, where responsible for the reduced lipid digestibility observed in our previous study (Grundy, Quint, Rieder, Ballance, Dreiss, Cross, et al., 2017). The incubation times were selected because 1 h corresponds to the duration of the duodenal phase used in our digestion model and 72 h is the time at which the maximum of β-glucan released from the oat matrices was recorded (Grundy, Quint, Rieder, Ballance, Dreiss, Butterworth, et al., 2017).

**Fig. 1:**
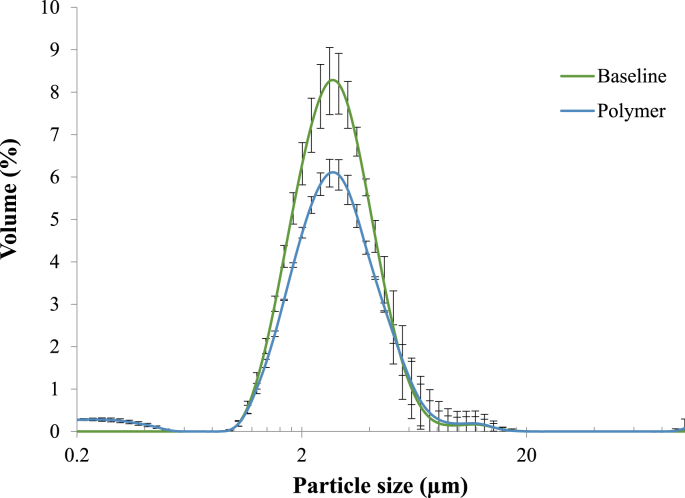
Particle size as percentage volume of the emulsion at baseline () and in presence of the polymers (). Values are presented as means ± SD (n = 3).

To make the oil in water emulsions, sunflower oil was added to 1% (w/w) WPI solution to obtain a 6.4 wt% oil solution as previously reported (Grundy, Quint, Rieder, Ballance, Dreiss, Cross, et al., 2017). Briefly, the emulsion premix was homogenized (Ultra-Turrax T25, IKA^®^ Werke, from Fisher Scientific Ltd.) at 11,000 rpm for 1 min. The pre-emulsion was then sonicated with an ultrasonic processor (Sonics & Materials Inc, Newtown, USA) at 70% amplitude for 2 min. The particle size distribution of the emulsions with and without polymers were measured with a laser diffraction instrument (LS13320^®^, Beckman Coulter Ltd., High Wycombe, UK), the average droplet size (d_32_) of both type of emulsions was 2.0 μm (Fig. 1). Emulsion samples (10.64 mL) were added to aqueous solutions containing either pure polymers or oat extracts (20 mL); the final concentration of the pure polymer mixtures ranging from 0.025 to 1.0% (w/v). The resulting mixtures were stirred at room temperature for 30 min before further analysis.

### 2.3 Rheological measurements

Rheological measurements (oscillatory and viscometry) of the emulsion and polymer samples were carried out with a dynamic rheometer (Advanced Rheometer AR 2000, TA instrument, Herts, UK) equipped with a conical concentric cylinder geometry (inner radius of 15 mm, cylinder length of 42 mm and gap of 2 mm) and a temperature-controlling Peltier unit. The measurements were run using controlled strain mode.

First, the linear viscoelastic region of each sample was determined with a strain sweep - 0.01–100% - at a fixed angular frequency of 6.28 rad s^−1^. Data collection started after temperature equilibration of 2 min. Then, the storage (G′) and loss (G″) moduli were measured at 25 °C by a dynamic frequency sweep conducted over an angular frequency range between 0.1 and 1000 rad s^−1^ and at a constant strain of either 5 or 10% depending on the linear viscoelastic region of the sample formerly determined. Viscosity flow curves were obtained in duplicate at 25 °C after 2 min temperature equilibration with the operating shear rate ranging from 0.01 to 1000 s^−1^ with seven measurement points per decade.

### 2.4 Microstructural analysis

Aqueous solutions containing pure polysaccharides and oat extracts, with or without emulsion, were visualised, immediately after preparation as described in Section 2.2., using an optical (Olympus BX60, Olympus, Southend-on-Sea, UK) or a confocal laser scanning (CLSM; Zeiss LSM 780 confocal microscope, Carl Zeiss Ltd, Cambridge, UK) microscopes. For the CLSM, Nile red (1 mg/mL in dimethyl sulphoxide) and calcofluor white (2% w/v in deionised water) were used to identify the lipids and the β-glucan, respectively. The images were captured using a 40 × (N.A. 1.2) objective lens. The samples were excited using an argon laser at 488 nm (Nile red) and 405 nm (calcofluor white), the fluorescence emitted by the samples was detected at 570–650 nm (Nile red) and 406–460 nm (calcofluor white).

### 2.5 Emulsion stability analysis

The stability of the emulsion alone or with the polymers (freshly prepared as described in Section 2.2.) was monitored visually by taking photographs of the mixture at different time points (0, 1, 2, 4, 6 and 24 h; see Fig. S1 of the supplementary material). Additionally, fluctuation in the stability of the emulsion and emulsion/polymer (pure polymer and incubation liquid) mixtures were measured using a laser scanning instrument (Turbiscan Lab Expert analyser, Formulaction SA, Toulouse, France). Twenty mL of a freshly prepared sample were placed into a flat-bottomed cylindrical glass cell and scanned at 10 min interval for 6 h to determine the light scattered from the emulsions as a function of the height (40 μm intervals) of the sample. The intensity of the scattered light gives information on flocculation, creaming and sedimentation in the emulsion. All stability experiments were performed at room temperature and at least in duplicate.

### 2.6 Theoretical prediction of thickening and flocculation

#### 2.6.1 Thickening

The ability of a polymer molecule to increase the viscosity of aqueous solutions can be described by the following relatively simple expression (McClements, 2000):

(1) $$\frac{\eta }{\eta_{1}}={\left(1-\frac{\varphi_{E}}{\varphi_{C}}\right)}^{-2}$$

In this equation, η is the apparent shear viscosity of an aqueous polymer solution, η_1_ is the viscosity of water, φ_E_ is the effective volume fraction of the polymer molecules, and φ_C_ is the critical packing fraction of the polymers (≈0.57). The critical packing fraction represents the polymer concentration where polymer molecules become so closely packed together that the solution viscosity increases steeply. The effective volume fraction of the hydrated polymer molecules can be estimated using the following expression:

(2) $$\varphi_{E}=\frac{4}{3}\pi R_{h}^{3}\left(\frac{cN_{A}}{M}\right)$$

here, *R*_*h*_ is the hydrodynamic radius of the polymer molecules (m), *c* is the polymer concentration (kg m^−3^), *N*_A_ is Avogadro's number (6.02 × 10^23^ mol^−1^), and *M* is the Mw of the polymer molecules (kg mol^−1^). These equations predict that the solution viscosity should increase as the polymer concentration and *R*_*h*_ increase, and the Mw decreases. It should be noted that the *R*_*h*_ and Mw are actually dependent on each other, with *R*_*h*_ increasing as Mw increases, which has to be taken into account. Moreover, these equations assume that the polymer molecules are monodisperse hard spheres, whereas in reality they are polydisperse soft spheroids. Nevertheless, they still provide some useful qualitative insights into the factors affecting solution rheology.

A critical viscosity concentration (CVC) can be estimated from the above equations, *i.e.,* the polymer concentration where the viscosity increases steeply due to overlap of the polymer molecules:

(3) $$CVC=\frac{3\varphi_{E}^{*}M}{4\pi R_{h}^{3}N_{A}}$$

As described previously (Bai et al., 2017), it is assumed that the CVC corresponds to the polymer concentration where the viscosity of the polymer solution is hundred times greater than that of pure water (η/η_1_ = 100). The value was selected because a 100-fold increase in solution viscosity will greatly retard the creaming of the droplets in emulsions. Inserting η/η_1_ = 100 and φ_C_ = 0.57 into Equation (1) leads to the following expression for the effective polymer volume fraction where overlap occurs: φ^∗^_E_ ≈ 0.53. Then, inserting this value into Equation (3), gives the expression: CVC = 21 × *M*/*R*_*h*_^3^ (wt%) when *M* is expressed in kg mol^−1^ and *R*_*h*_ is expressed in nm. The smaller the magnitude of CVC, the greater is the effectiveness of the polymer at increasing the solution viscosity.

#### 2.6.2 Depletion flocculation

The ability of a non-adsorbed polymer to promote depletion flocculation depends on its effectiveness at increasing the attractive osmotic forces between oil droplets (Jenkins & Snowden, 1996). As discussed in the Introduction section, this osmotic attraction occurs because the polymer molecules are excluded from a narrow depletion zone around each droplet, which leads to a concentration gradient in the system. The volume of the depletion zone can only be reduced when two or more droplets come into close proximity. The attractive interaction between two droplets brought into contact through this effect can be estimated using the following expression (McClements, 2000):

(4) $$W_{Dep}=\frac{-2\pi kTN_{A}R_{h}^{2}c}{M}\left(1+\frac{2\pi R_{h}^{3}cN_{A}}{3M}\right)\left(R+\frac{2}{3}R_{h}\right)$$

here *W*_Dep_ is the depletion attraction between the droplets, *k* is Boltzmann's constant, *T* is the absolute temperature, and *R* is the oil droplet radius. The majority (>98%) of oil droplets in an emulsion flocculate when the depletion attraction is stronger than about −4 kT, which allows a critical flocculation concentration (CFC) to be defined (Bai et al., 2017):

(5) $$CFC=\frac{-b\pm \sqrt{b^{2}-4a}}{2a}$$

where,

(6) $$b=\frac{-\pi N_{A}R_{h}^{2}}{2M}\left(R+\frac{2}{3}R_{h}\right)$$

(7) $$a=b\left(\frac{2\pi R_{h}^{3}N_{A}}{3M}\right)$$

### 2.7 *In vitro* duodenal digestion

The oral and gastric phases were omitted in the present study as our main aim was to study the starting material in a less dilute system and apply a simple model in order to understand in more detail the processes occurring in the duodenal phase. In our view, the important aspect that needed to be taken into consideration in the present experiments was to keep the conditions (pH, digestive agents and source of lipid: the emulsion) identical across the range of materials to permit a fundamental understanding of the mechanisms controlling emulsion stability and digestion.

The kinetics of release of FFA during *in vitro* duodenal digestion was measured in a pH-stat vessel. Nine mL of the incubation solution containing the polymer was added to 10 mL of sunflower oil emulsion, 15 mL of bile solution, 1 mL of NaCl, 1 mL of CaCl_2_, and 1.5 mL of pancreatin solution (digestion) or phosphate buffer (blank). The details about the preparation of the digestion reagents are presented in (Grundy et al., 2015) (Grundy, Wilde, Butterworth, Gray, & Ellis, 2015). The rate and extent of FFA released during lipolysis of the sunflower oil emulsion were monitored by titration with 0.10 M NaOH for 60 min at 37 °C, pH 7 using a pH-stat (KEM AT-700, Kyoto Electronics Manufacturing Co., Ltd., Kyoto, Japan). All lipolysis experiments were carried out in triplicate.

The digestibility experiments could not be performed with the pure polymer samples given that their high viscosity interfered with pH measurements. Indeed, we experienced issues in rapidly reaching a steady-state pH due to a delay in mixing therefore adjustment of the pH, and the pH measurements were highly variable between replicates (*data not shown*).

### 2.8 Statistical analysis

The data were analysed using SPSS version 22.0. For all tests, the significance level was set at p < 0.05 (2 tailed) and the data were expressed as means of duplicates or means of triplicates ± standard deviations. The differences between the lipolysis of emulsion alone and in presence of oat extracts were analysed by one-way analysis of variance (ANOVA) followed by Dunnett's post-hoc test.

## 3 Results and discussion

In a recent study, we showed that the tendency for depletion flocculation to occur in sunflower oil-in-water emulsions, and its impact on the rate and extent of lipid digestion, was not directly related to the concentration of β-glucan in the reaction system (Grundy, Quint, Rieder, Ballance, Dreiss, Cross, et al., 2017). In the present work, we therefore aimed to make a more detailed investigation of the impact of polysaccharide properties on emulsion stability and lipid digestion using water-soluble polysaccharides with well-defined molecular characteristics and water-soluble extracts isolated from oats (flakes, flour and bran).

### 3.1 Material characterisation

The pure polymer samples had a polysaccharide (galactomannan or β-glucan) concentration between 87.1 and 93.8% dry-weight basis (d.b.), and the weight-average Mw of these polysaccharides ranged from 220 to 1020 kg mol^−1^ as measured by SEC-MALLS (Table 1). The amount of β-glucan contained in the complex oat materials was lower than the purified samples: 5% d.b. in the oat flakes/flour and 37.9% d.b. in oat bran. The lipid content of the flakes/flour were also markedly different from the oat bran, 10.8 and 3.8% d.b., respectively, whereas BG1, BG2 and BG3 did not contain any lipid. The Mw of the β-glucan extracted from the oat flakes and flour were both around 1700 kg mol^−1^ but the one from the oat bran was higher (2100 kg mol^−1^). The weight-average *R*_*h*_ of the pure polysaccharides in solution ranged from around 23 to 78 nm, and increased with increasing Mw.

**Table 1:** Chemical composition of the purified polymers (guar gum and β-glucans) and oat materials (oat bran, flour and flakes), weight-average molecular weight (Mw), weight-average hydrodynamic radius (*R*_*h*_), critical viscosity concentration (CVC), and critical flocculation concentration (CFC) of the polymers. The composition data are expressed on a dry weight basis.

	Moisture (%)	Crude lipid (%)	β-glucan (%)	Galactomannan (%)	Mw (kg mol^−1^)	*R*_*h*_ (nm)	CVC (%)	CFC (%)
Guar gum	10.2	1.1	–	87.1	2490	78	0.11	0.037
BG1	8.1	–	90.1	–	1020	51	0.18	0.040
BG2	14.6	–	91.0	–	650	38	0.24	0.044
BG3	4.0	–	93.8	–	272	23	0.38	0.042
Bran	8.3	3.8	37.9	–	2100	78		
Flakes	10.8	10.8	5.0	–	1680	70		
Flour	10.8	10.8	5.0	–	1740	70		

### 3.2 Impact of polymer type on rheology

**Fig. 2:**
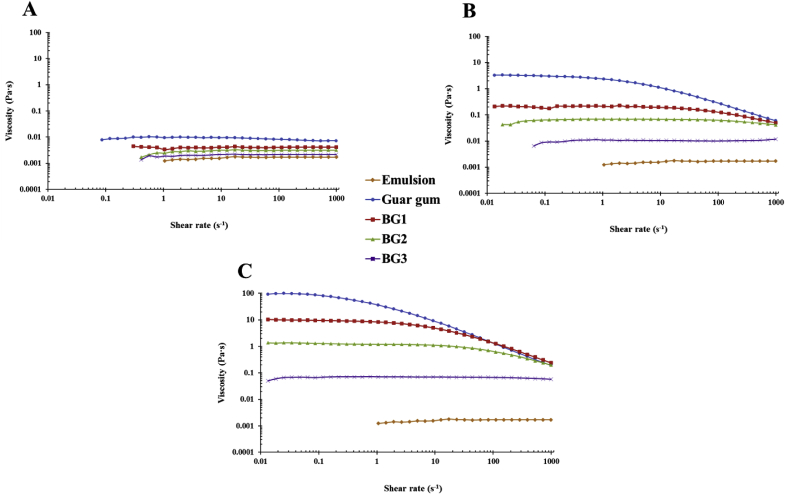
Log-log plot of steady shear viscosity versus shear rate for emulsion and polymer (guar gum and β-glucan) mixtures at concentration of 0.1 (A), 0.5 (B) and 1.0% (C). BG1 corresponds to high Mw β-glucan, BG2 to medium Mw β-glucan, and BG3 to low Mw β-glucan.

The measured flow curves of the emulsion/polymer mixtures (Fig. 2) were similar to those reported for the polymer solutions alone (Grundy, Quint, Rieder, Ballance, Dreiss, Butterworth, et al., 2017). As anticipated, the apparent viscosity increased with both concentration and Mw. Indeed, solutions with the lowest polysaccharide concentration and Mw (0.1% BG3) had the lowest viscosity, whereas those with the highest concentration and Mw (1.0% guar gum) had the highest viscosity over the range of shear rates. The increase in viscosity with increasing Mw can be attributed to the fact that larger polysaccharides occupy a greater effective volume (polymer chain + water) (Section 3.6.). Apart from BG3, the 1.0% polysaccharide solutions had a plateau region at low shear rates, followed by a shear-thinning region at higher shear rates, which is characteristic of semi-flexible polysaccharides or entangled polymers (Ellis, Wang, Rayment, Ren, & Ross-Murphy, 2001). The 0.1% polymer solutions all behaved as Newtonian fluids, i.e., the viscosity was independent of shear rate in so-called dilute solution conditions. This suggests that the polysaccharide molecules were not entangled in these solutions, i.e., the polymer concentration was below the critical overlap concentration (c*).

**Fig. 3:**
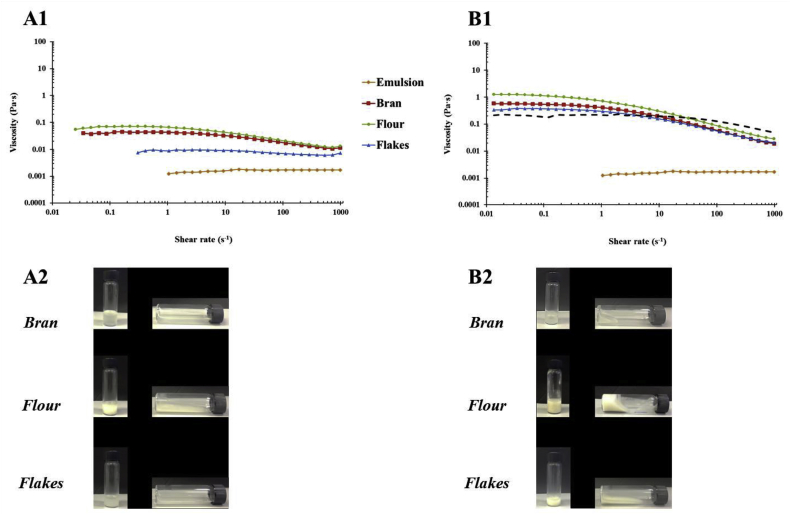
Log-log plot of steady shear viscosity versus shear rate for emulsion mixed with oat bran, flour and flakes solutions collected after 1 h (A) and 72 h (B) of incubation. Pictures A2 and B2 illustrate the collected incubation solutions before adding the emulsion. The dashed line on Fig. B1 represents the flow curve of BG1 at 0.5% (similar concentration than the β-glucan contained in the oat extracts).

For the flakes after 1 h of incubation, the viscosity appeared Newtonian over the range of shear rates (Fig. 3A1) suggesting the β-glucan concentration (0.1% w/v, Table 2) was not high enough to achieve polymer entanglement. Whereas both the bran and flour samples, displayed some shear thinning. Despite containing fairly similar β-glucan concentrations (0.26–0.27%, Table 2), the oat extract solutions isolated from the flour after 1 h of incubation had higher apparent viscosity than those isolated from the oat bran across the whole range of shear rates studied (Fig. 3). This phenomenon may have been because a higher level of starch was released from the oat flour than from the bran (see Fig. 7). Both the flour and bran extracts had much higher viscosities than the oat flakes extract, which can be attributed to the lower amounts of β-glucan and starch released from the flakes. The incubation time of the oat flakes, flour and bran also impacted their viscosities. Therefore, the samples that were incubated for 72 h had higher apparent viscosity than those incubated for 1 h, which is a result of a larger quantity of polymers (e.g., β-glucan, starch, and proteins) being released after prolonged incubation (Table 2 and Fig. 3, Fig. 7). The addition of the emulsion appeared to have diminished the disparities that existed in the viscosity profiles between the flakes/flour and the bran (Grundy, Quint, Rieder, Ballance, Dreiss, Butterworth, et al., 2017). The reason for this could be the swelling of the starch when in presence of additional liquid (i.e. the emulsion). The viscosity profiles of the 72 h incubation samples were fairly similar, and were consistent with the concentrations of β-glucan released (Table 2) compared to the pure polymer with the highest Mw (∼1020 kg mol^−1^) and equivalent concentration (∼0.5%), i.e., BG1. This implies that β-glucan was the main oat component affecting the viscosity of those samples.

**Fig. 4:**
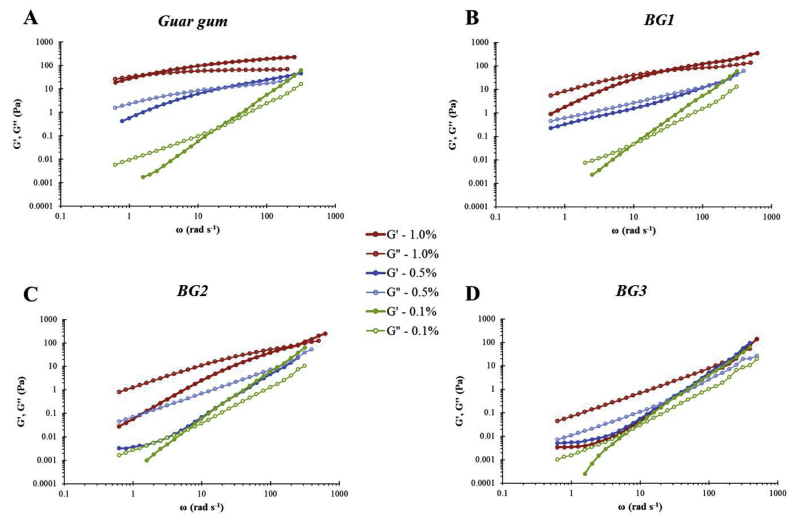
Log-log plot of storage (G′) and loss (G″) moduli versus angular frequency for mixtures of emulsion and pure polymer at different concentrations (0.1, 0.5 and 1.0% in final preparation). BG1 denotes high Mw β-glucan, BG2 medium Mw β-glucan, and BG3 low Mw β-glucan.

**Fig. 5:**
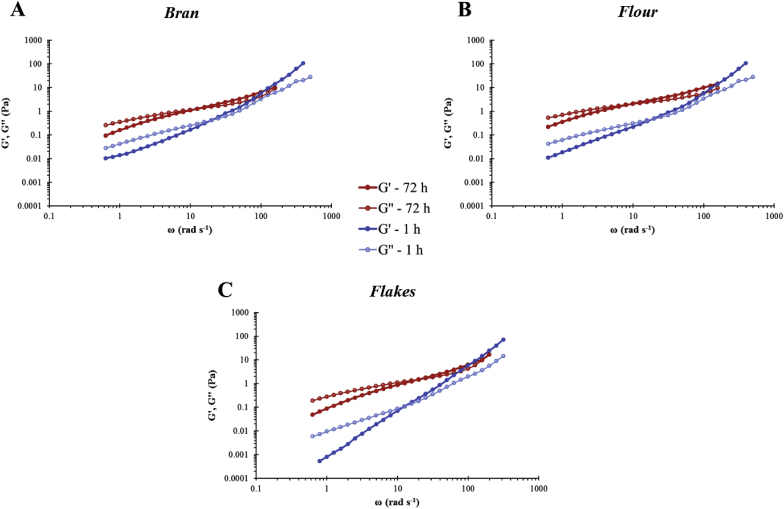
Log-log plot of storage (G′) and loss (G″) moduli versus angular frequency for emulsion mixed with oat bran (A), flour (B) and flakes (C) solutions collected after 1 h and 72 h of incubation.

**Fig. 6:**
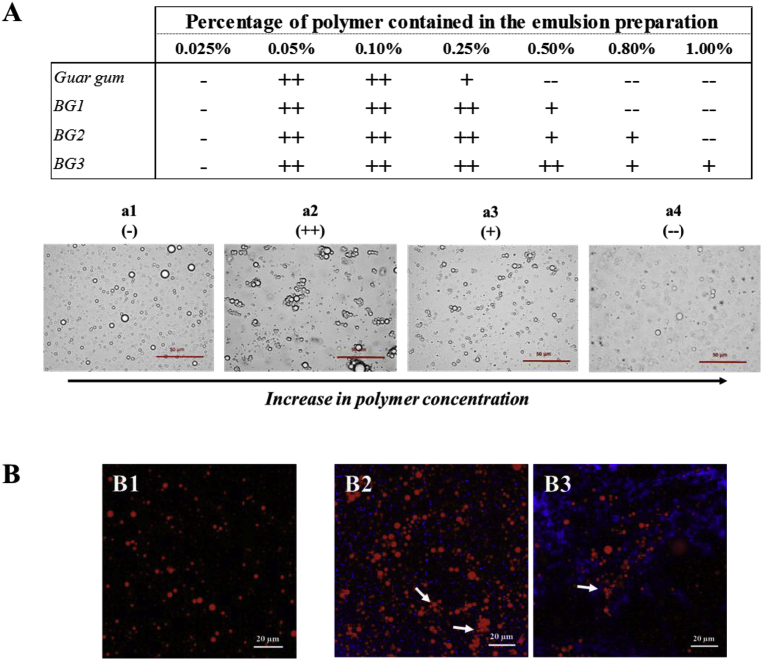
Impact of pure polymers on emulsion stability: summary table of the flocculation observed at different concentrations of the polymers using light (A) and confocal (B) microscopy. BG1 corresponds to high Mw β-glucan, BG2 to medium Mw β-glucan, and BG3 to low Mw β-glucan. Note in figures B2 and B3 the β-glucan stained in blue surrounding the oil droplets (red) creating depletion flocculation (white arrows). Four flocculation regimes were defined: a1 (−) no flocculation, a2 (++) extensive flocculation, a3 (+) limited flocculation, a4 (--) high viscosity prevents the movement of the droplets, no flocculation was observed. (For interpretation of the references to colour in this figure legend, the reader is referred to the Web version of this article.)

**Fig. 7:**
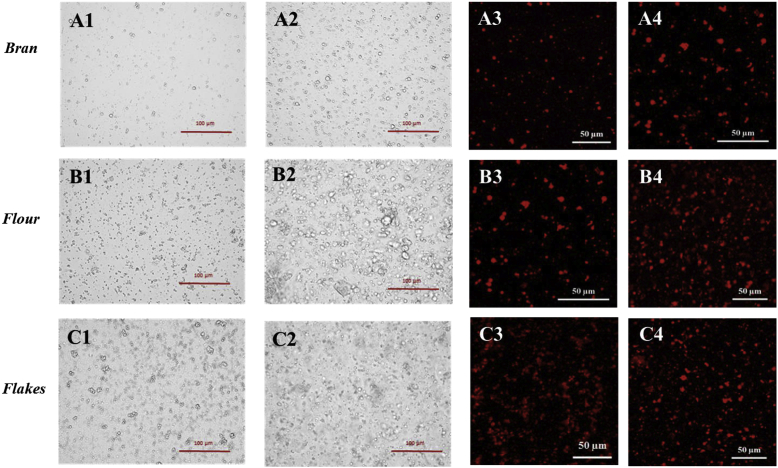
Light (1 and 2) and confocal (3 and 4) microscopy images of emulsion mixed with oat bran (A), flour (B) and flakes (C) solutions collected after 1 h (1 and 3) and 72 h (2 and 4) of incubation. The oil droplets are stained with Nile red (3 and 4). (For interpretation of the references to colour in this figure legend, the reader is referred to the Web version of this article.)

**Table 2:** Concentrations, in percentage, of the β-glucan present in the solutions collected from the incubation of oat materials (bran, flakes and flour).

	Concentration of β-glucan in incubation extract (%)	
	*1 h*	*72 h*
Bran	0.27	0.54
Flour	0.26	0.57
Flakes	0.10	0.51

Oscillatory measurements were performed to describe the viscoelastic properties of the samples, which provided insights into the possible conformation of the polysaccharides in solution. For all concentrations, the guar gum and BG1 samples exhibited a crossover point between G′ and G″ indicating that these polymers formed an entangled network in solution and showed an elastic-like behaviour at higher frequencies (Fig. 4), which is in agreement with previous studies (Agbenorhevi, Kontogiorgos, Kirby, Morris, & Tosh, 2011; Ren, Ellis, Ross-Murphy, Wang, & Wood, 2003; Robinson, Ross-Murphy, & Morris, 1982).

The oat materials, for both incubation times, showed similar trend in G′ and G″ profiles across the range of angular frequencies, although the 1 h flake sample had lower moduli than the corresponding values for bran and flour (Fig. 5), due to the lower amounts of β-glucan released (Table 2). The general pattern of the plots indicates that the β-glucan molecules released, above a concentration of 0.1%w/v, overlapped and generated an entangled network (Morris, 2001). These data are consistent with the amount of β-glucan presents in solution in the oat samples (Table 2).

### 3.3 Impact of polymer type on emulsion stability

The susceptibility of the emulsions to droplet flocculation was initially determined by optical and confocal fluorescence microscopy (Fig. 6). Droplet flocculation was only observed at certain concentrations for the different polymers. The “stability pattern” appeared to be similar for all polymers but the concentrations at which the depletion flocculation occurred differed (Fig. 6A). In general, the emulsions were stable to flocculation at low polymer concentrations, unstable at intermediate concentrations, and stable again at high polymer concentrations. For the purpose of clarity, we defined four different flocculation regimes as the polymer concentration was increased. Firstly, the addition of low concentrations of polymer did not disturb the emulsion and it appeared similar to the control emulsion (a1). Second, as the polymer concentration increased, extensive flocculation occurred (a2). Third, when the polymer concentration was increased further, a more limited amount of flocculation was observed (a3). Fourth, high concentrations of polysaccharides did not induce flocculation and the emulsion was stable (a4). For all polymers no flocculation was observed at 0.025%, and strong flocculation (a2) was observed at 0.05%. As the polysaccharide Mw decreased, the concentration range over which flocculation was observed increased: guar gum < BG1 < BG2 < BG3.

Several images were taken throughout the sample to visually assess the degree of flocculation. Fig. 6B shows representative images of stable (Fig. 6B1) and flocculated emulsions (Fig. 6B2 and B3). Selective fluorescence staining indicated that the β-glucan (stained blue) was present in the aqueous phase surrounding the oil droplets (stained red), which is strong evidence for the ability of this polysaccharide to promote flocculation through a depletion mechanism. The particle size distributions of the emulsion alone (baseline) and in presence of the polymers were virtually identical (average size of 2.0 μm as shown in Fig. 1). During the laser diffraction measurement, the sample was diluted (up to 1000 fold) when loading the sample into the instrument. This demonstrated that the droplet aggregation was reversible and depletion flocculation is therefore likely to be the phenomenon occurring in our systems. These findings confirm that β-glucan is a non-absorbing polymer under those conditions, i.e., pH 7 and emulsion stabilised by whey proteins, as recently reported (Zielke, Lu, Poinsot, & Nilsson, 2018). The β-glucan also formed aggregates at certain Mw and concentrations (a2 (++)), which is a well-known phenomenon (Agbenorhevi et al., 2011; Doublier & Wood, 1995; Lazaridou, Biliaderis, & Izydorczyk, 2003; Li, Cui, Wang, & Yada, 2011). On the other hand, a three-dimensional network of aggregated polysaccharides appeared to form at the highest polymer concentrations used, which immobilised the oil droplets and prevented them from flocculating (Fig. 6A a4). Indeed, above the critical concentration (c*) and Mw of the galactomannan or β-glucan, when entanglement occurs, and a visco-elastic network is generated, the oil droplets are less able to diffuse through the entangled network and therefore the droplets cannot approach each other, and their depletion zones cannot overlap. This is possible as once an entangled network is formed, it is capable of arresting microscopic phase separation (McClements, 2000).

The optical and confocal microscopy images of emulsions mixed with the water-soluble extracts isolated from the oat bran, flour, and flakes indicated that they contained various types of colloidal particles (Fig. 7). These were probably oil droplets from the emulsions, as well as starch granules and protein aggregates that leached out of the oat materials. The microscopy images also indicated that more compounds were released after 72 h than 1 h of incubation, especially for the oat flour, which is consistent with the viscosity data (Fig. 3) and our former work (Grundy, Quint, Rieder, Ballance, Dreiss, Butterworth, et al., 2017). Moreover, the confocal microscopy images clearly show that the oil droplets were flocculated in a number of the samples, *i.e.,* the oat flour extracts (72 h) and the oat flake extracts (1 h and 72 h) (Fig. 7B4, C3, and C4). This suggests that the extracts contained water-soluble compounds/particulates capable of promoting depletion flocculation of the oil droplets in the emulsions.

### 3.4 Stability of the emulsions in presence of pure polysaccharides

**Fig. 8:**
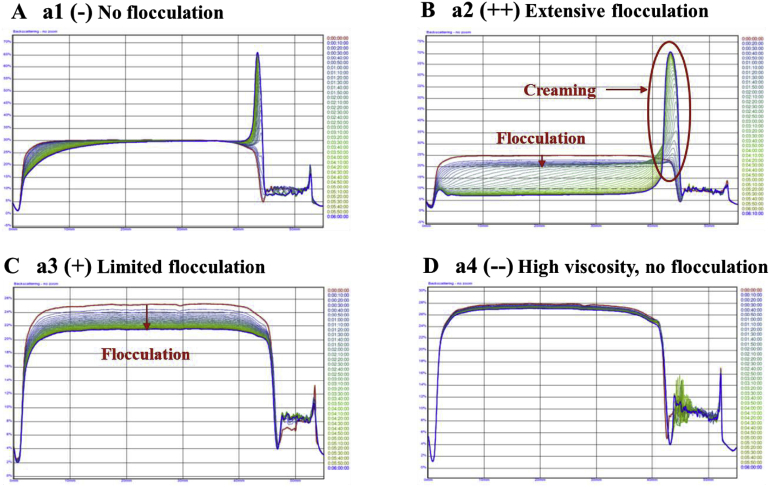
Typical trends of the light-scattering curves for mixtures of emulsion and pure polymer at different concentrations. BG1 denotes high Mw β-glucan, BG2 medium Mw β-glucan, and BG3 low Mw β-glucan. Four flocculation regimes were defined: a1 (−) no flocculation, a2 (++) extensive flocculation, a3 (+) limited flocculation, a4 (--) high viscosity prevents the movement of the droplets, no flocculation was observed.

Further information about the stability of the emulsions to creaming and flocculation was obtained by measuring the backscattering versus height profiles using a laser scanning instrument. Typical creaming profiles describing the different observed stability states are shown in Fig. 8. The X-axis denotes the distance or height from the sample base, and the Y-axis denotes the backscatter intensity, a function of the number and size of scattering particles or droplets. Creaming is denoted by a reduction in intensity at the left-hand side of the graph (base of the sample) with a concomitant increase in the intensity at the right-hand side (top of the sample) (Fig. 8A). Flocculation is denoted by a decrease in the intensity of the backscattering at the centre of the emulsion (Fig. 8B and C), due to the fact that the droplets move closer together and therefore scatter light less strongly as a consequence of the reduced number of scattering centres. The backscattering profiles of the polysaccharides-emulsion mixtures were consistent with the microscopy images (Fig. 8), with the different polymers exhibiting similar general trends. Again, the backscattering profiles could be separated into four different categories depending on polymer concentration. First, no flocculation occurred at lower polymer levels, but a small amount of creaming was observed, i.e., the backscattering intensity in the central part of the sample remained constant, but there was a slight increase at the top and decrease at the bottom (Fig. 8A). This behaviour can be attributed to the upward movement of the individual oil droplets since they have a lower density than the surrounding aqueous phase. Second, as the polymer concentration increased, the mixture became highly unstable to flocculation and creaming, i.e., the backscattering intensity in the central region decreased appreciably, and there was a large increase in intensity at the top of the sample. This effect can be attributed to the fact that flocculation leads to an increase in particle size, which promotes gravitational separation, and that the viscosity is not high enough to inhibit the creaming of oil droplets (Mengual, Meunier, Cayre, Puech, & Snabre, 1999). Third, a further increase in polymer concentration led to flocculation without creaming, i.e., there was a decrease in the backscattering intensity in the central region, but little change at the top or bottom of the sample (Fig. 8C). This effect can be attributed to the fact that the non-adsorbed polymers induced depletion flocculation, but the aqueous phase viscoelasticity was sufficient to stop the creaming of the flocculated droplets. Fourth, at high polymer levels, neither flocculation nor creaming was observed, i.e., the backscattering profile remained constant during storage (Fig. 8D). Indeed, the movement of flocs will be hindered by an increase in viscosity of the aqueous phase up to the point where the viscosity was high enough that it restricted the movement of individual oil droplets, so they could not approach each other. This prevented droplet flocculation, despite the high depletion forces generated by the polymer (Bai et al., 2017; McClements, 2000; Syrbe et al., 1998).

### 3.5 Impact of oat extracts on emulsion stability

**Fig. 9:**
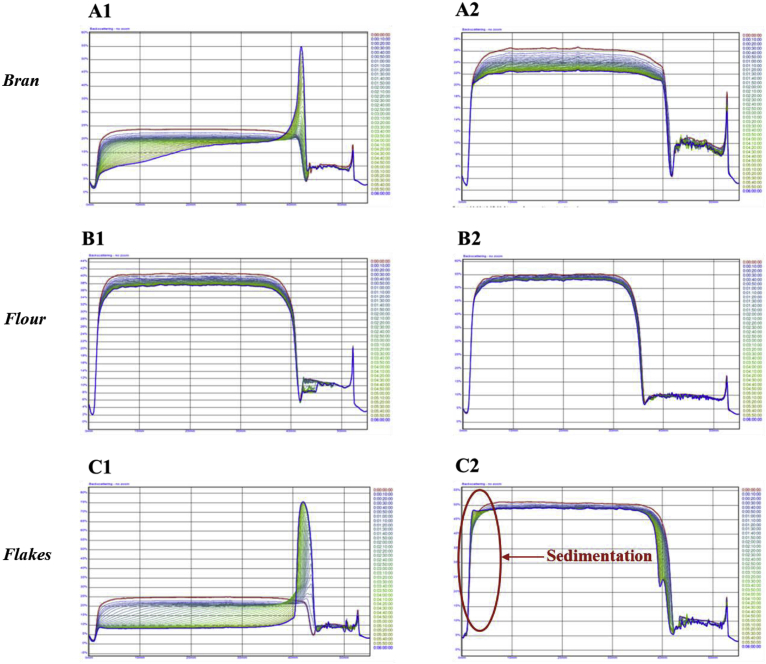
Light-scattering curves for emulsion mixed with oat bran (A), flour (B) and flakes (C) solutions collected after 1 h (1) and 72 h (2) of incubation.

The laser scanning technique was also used to study the impact of the oat extracts on the physical stability of the emulsions (Fig. 9). These samples displayed a more complex behaviour than the purified polysaccharide solutions. When the emulsion was mixed with the solution containing the oat flake extracts, the droplets flocculated to a greater extent than for the solutions containing either the flour and bran extracts (as seen in Fig. 7), with creaming starting fairly rapidly (after ∼20–30 min). The 1 h bran extract also exhibited some creaming but at a later stage (after 2 h, Fig. 9A2). Therefore, the concentration of water-soluble extracts in the solutions increased as the extraction time increased, which will promote the tendency for depletion flocculation to occur. No creaming was detected for any of the 72 h samples, however sedimentation occurred for the oat flake extracts (Fig. 9C2). This was indicated by a decrease in intensity at the top of the sample, together with an increase in intensity at the base of the sample. Similarly to the processes occurring with pure polymers, the high viscosity of the oat flake and bran extract solutions, as shown in Fig. 3B1 and 3B2, is likely to have hindered the mobility of the oil droplets. For the extracts, there was some evidence of an increase in backscattering at the bottom of the samples after prolonged storage, which was attributed to the sedimentation of dense particulates, such as starch granules, cell fragments, and protein aggregates from the oat material.

### 3.6 Theoretical prediction of thickening and flocculation

The ability of polymer molecules to thicken emulsions and to promote depletion flocculation has been theoretically related to their molecular characteristics (Bai et al., 2017; McClements, 2000). These models can be used to understand the impact of polysaccharide concentration and Mw on emulsion stability.

The CVC values were calculated from equation (3) for the four polysaccharides used in this study from their known mean Mws and hydrodynamic radii (Table 1). The values of CVC increased in the following order: guar gum (0.11%) < BG1 (0.18%) < BG2 (0.24%) < BG3 (0.38%). These calculations suggest that a higher concentration of BG3 is needed to increase the viscosity than guar gum, which is in agreement with the experimental measurements (Fig. 3).

The CFC of the different polysaccharides used in this study were calculated from the equations described in Section 2.6.2. (Table 1). The values of the CFC decreased in the following order: guar gum (0.037%) < BG1 (0.040%) < BG2 (0.044%) < BG3 (0.042%). Thus, a lower concentration of guar gum should be required to promote depletion flocculation than BG3. Interestingly, the CFC values are higher than the CVC values for the higher Mw polymers (guar gum and BG1), which suggests that the viscosity of the emulsions would be relatively high before the oil droplets flocculated. Conversely, the CFC values are lower than the CVC values for the lower Mw polymers (BG2 and BG3), which confirmed that droplet flocculation and creaming occur at intermediate polymer concentrations, but creaming is hindered at higher polymer levels. These differences may account for the visual observations that the BG3 emulsions are unstable to flocculation and creaming over a wider range of polymer levels than the guar gum emulsions (Fig. 6A).

### 3.7 Impact of oat extracts on lipid digestibility

**Fig. 10:**
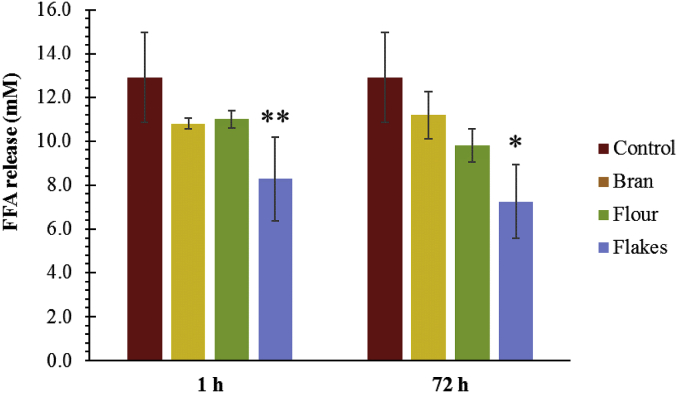
Amount of free fatty acids (FFA) released during the simulated duodenal digestion of emulsion alone (red) or in presence of solutions collected from the incubation of oat bran (yellow), flour (green) and flakes (blue). Statistical significance was determined using a one-way ANOVA (*p < 0.05 and **p < 0.01, bran or flour or flakes vs emulsion, n = 3). (For interpretation of the references to colour in this figure legend, the reader is referred to the Web version of this article.)

Finally, we examined the impact of the oat extracts from the oat flakes, flour, and bran on the extent of lipid digestion under simulated duodenal conditions. Fig. 10 shows that the extent of lipid digestion was significantly reduced for the flakes, for both 1 h (p = 0.001) and 72 h (p = 0.034) of incubation, but not for the flour and bran. However, longer incubation time of the flour appeared to decrease the amount of FFA formed during the lipolysis of the emulsion, albeit the difference was not significant. Overall, these results suggest that the oat extracts were able to inhibit lipid digestion (the kinetics of FFA production vs time can be found in Fig. S2 of the supplementary material). There are a number of possible reasons for this effect. First, an increase in the viscosity of the gastrointestinal fluids due to the presence of the dietary fibres may slow down the transport of lipase to the lipid droplet surfaces. The viscosity of the oat extract solutions decreased in the following order: flour > bran > flakes > control, and was higher for the 72 h extracts than for the equivalent 1 h extracts (Fig. 3). Thus, there did not seem to be a strong correlation between the viscosity of the solutions and the inhibition of lipid digestion for those systems. Second, an increase in droplet flocculation in the emulsions may have decreased lipid digestion by reducing the surface area of the lipid phase exposed to the lipase (Golding & Wooster, 2010). Based on β-glucan concentrations and the CFC of the pure polymers, the tendency for droplet flocculation to occur due to a depletion mechanism in the emulsions containing the different oat extracts should decrease in the following order (Fig. 9): bran > flour > flakes > control, which is not the case. By effectively excluding the first two possibilities, the third and most likely explanation is that there may be differences in the ability of the different extracts to bind components that are important in the lipid digestion process, such as bile salts, lipase, FFA, and calcium, which would alter the rate and extent of lipid digestion. In addition, the lack of a change in the initial lipolysis rate, but a change in the plateau of the FFA released (Supplementary Fig. S2) further suggests that β-glucan's role in lipid digestion is affected by the binding or entrapment of the products of digestion by the matrix.

The rate of hydration of the polysaccharides as well as the order in which the polymer network is formed and then mixed to the emulsion may also be critical to the functionality of oat and its compounds, in particular β-glucan (Veverka, Dubaj, Veverková, & Šimon, 2018; Wang, Ellis, & Ross-Murphy, 2008). The protocol used here, consisting of adding released compounds to an emulsion, seems like a realistic simulation of the course of events taking place during digestion in the human gastrointestinal tract. Indeed, it is likely that, during the digestion of oat or oat based food products, the lipids present in the stomach and small intestine would be emulsified (due to food processing or as a result of digestion) before becoming in contact with the β-glucan and viscous digesta since the β-glucan will first have to be released from the oat matrix (Grundy, Quint, Rieder, Ballance, Dreiss, Butterworth, et al., 2017).

## 4 Conclusions

The Mw and concentration of the polysaccharides studied influenced the stability of emulsions, such that lower Mws and concentrations resulted in depletion flocculation of the oil droplets stabilised with whey proteins. However, the observed effect was not linearly associated with the viscosity of the polymer solution. *In vitro* lipid digestion showed that oat flakes displayed the largest reduction in lipolysis, but this also did not relate directly with the solution viscosity.

Therefore, the mechanisms that leads to the reduction in blood lipid and cholesterol concentrations when oat is consumed still warrant further research. In particular, special attention should be put on the other compounds that are released during oat digestion and the interactions between them and with the oat matrix. Systematic characterisation of the interactions of the different oat extracts with lipid digestion components would be useful. Finally, additional work should be performed to identify if the depletion flocculation observed with the material presented in this investigation persists in the gastric compartment and succeeds in reducing the subsequent lipid digestion in the small intestine.
