# Supplementary material for: Influence of oat components on lipid digestion using an in vitro model: Impact of viscosity and depletion flocculation mechanism
Source: Food Hydrocoll. 2018 Oct;83:253–64. doi: 10.1016/j.foodhyd.2018.05.018 (PMC6020131; doi:10.1016/j.foodhyd.2018.05.018)

**Supplemental files**

**Fig S1.** Creaming pictures of pure polymers mixed with sunflower oil emulsion taken at 0, 1, 2, 4, 6 and 24 h. The red boxes highlight where creaming started to appear.


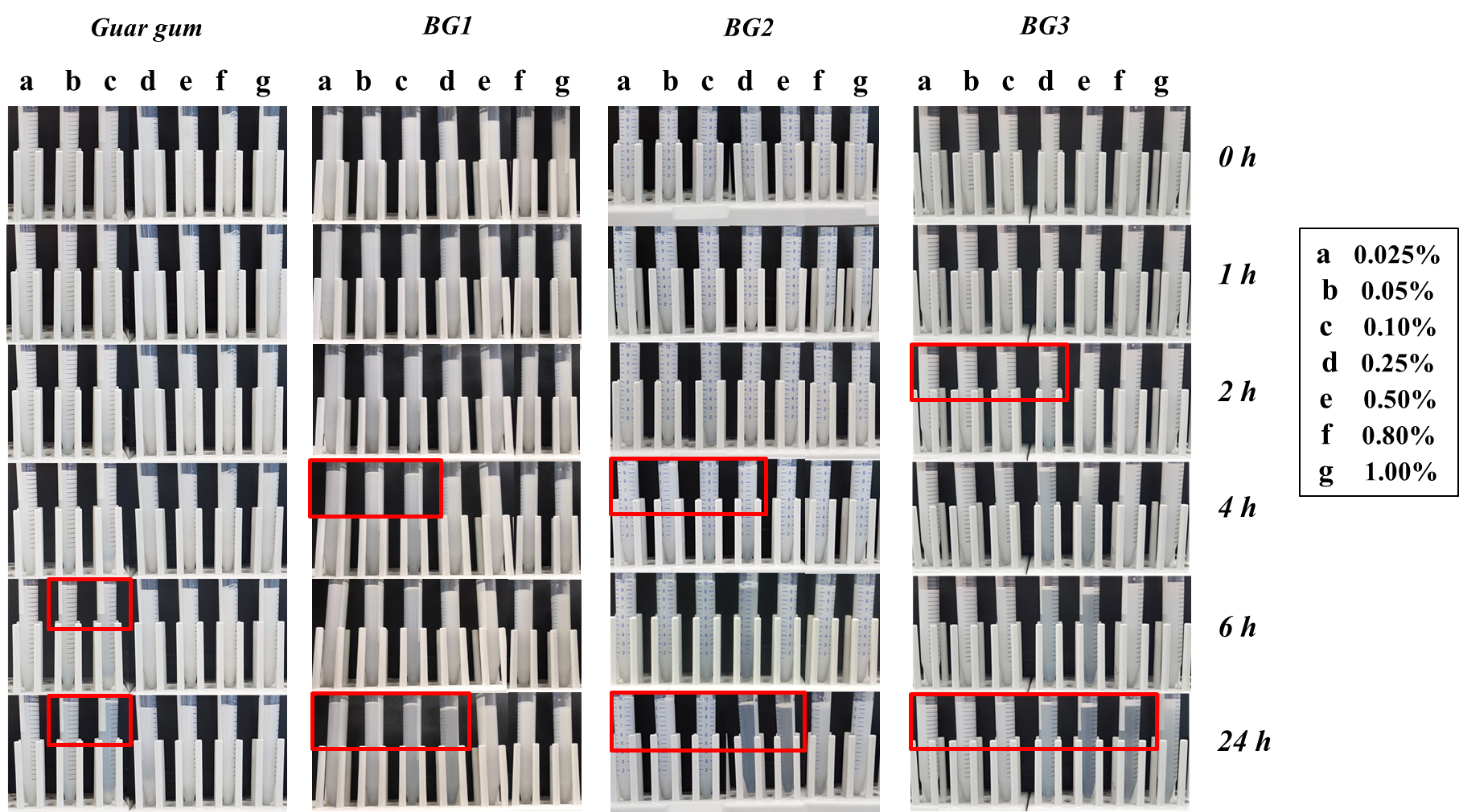


**Fig S2.** Kinetic curves of the amount of free fatty acids (FFA) released during the simulated duodenal digestion of emulsion alone (red) or in presence of solutions collected from the incubation of oat bran (yellow), flour (green) and flakes (blue).


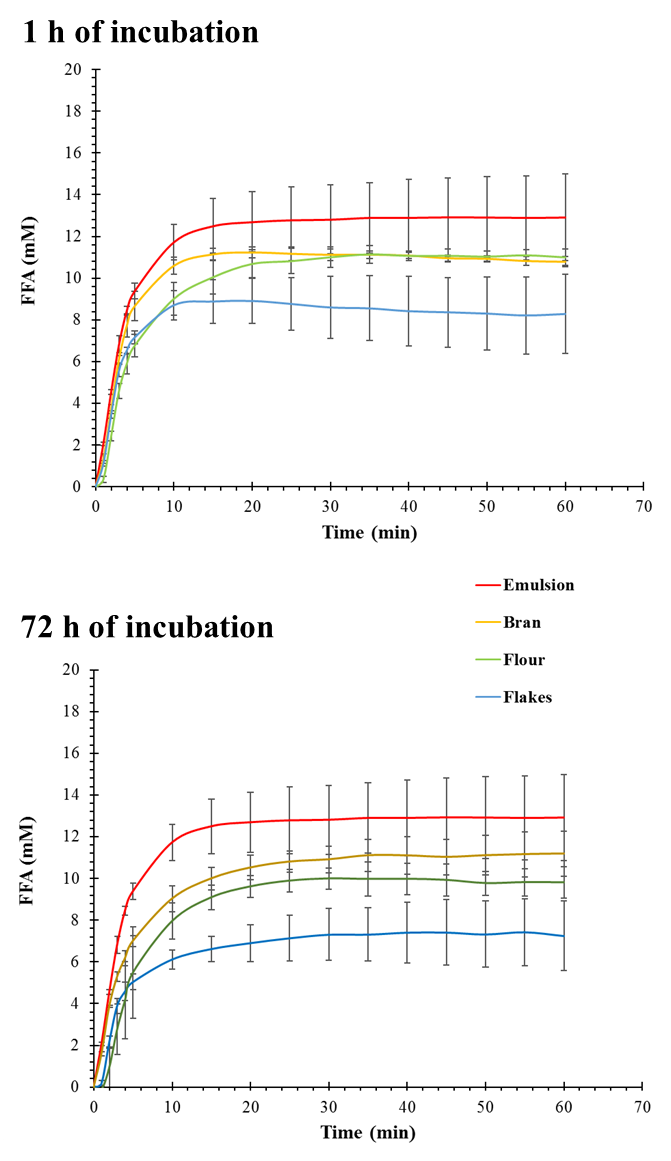

Supplement: Multimedia component 2 [file mmc2.docx]
